# Comparison of the Effect of EMLA™ Cream and the Valsalva Maneuver on Pain Severity during Vascular Needle Insertion in Hemodialysis Patients: A Controlled, Randomized, Clinical Trial

**DOI:** 10.1155/2022/8383021

**Published:** 2022-08-31

**Authors:** Hassan Babamohamadi, Zahra Ameri, Ilia Asadi, Mohammad Reza Asgari

**Affiliations:** ^1^Nursing Care Research Center, Semnan University of Medical Sciences, Semnan 3513138111, Iran; ^2^Department of Nursing, Faculty of Nursing and Midwifery, Semnan University of Medical Sciences, Semnan 3513138111, Iran; ^3^Student Research Committee, Semnan University of Medical Sciences, Semnan 3513138111, Iran

## Abstract

**Background:**

Pain due to vascular needle insertion has been reported in 40–60% of hemodialysis (HD) patients. Evidence suggests that there is typically no single method for relieving the pain of inserting vascular needles in HD patients. This study aimed to compare the effectiveness of EMLA cream and Valsalva maneuver (VM) on pain severity during vascular needle insertion in HD patients.

**Methods:**

This randomized, controlled, clinical trial was conducted on 90 patients undergoing hemodialysis in the hemodialysis unit of Kowsar Hospital, affiliated with Semnan University of Medical Sciences, in Semnan, Iran. Patients were selected via convenience sampling and were randomly assigned to one of the three groups (EMLA, VM, and control groups). For the patients in the EMLA group, 2.5 g of EMLA cream was applied 60 minutes before the start of dialysis. For patients in the VM group, a maneuver was performed for 16–20 seconds before the needle was inserted. Patients in the control group received only routine care without any additional interventions. The pain severity in the three groups was measured using the visual analog scale (VAS) two minutes after vascular needle insertion.

**Results:**

The results showed that the mean pain severity during cannulation was 2.06 ± 2.19 in the EMLA group, 3.2 ± 30.42 in the VM group, and 6.20 ± 1.49 in the control group, suggesting a significant difference between the groups (*P* < 0.001). Pairwise comparison of the mean pain severity showed that it differed significantly in the EMLA and VM groups from the control group (*P* < 0.001), but no significant difference was found between the EMLA and VM groups (*P*=0.067).

**Conclusion:**

According to the results, EMLA cream was as effective as VM in reducing the pain severity caused by arteriovenous fistula (AVF) cannulation. Therefore, the use of EMLA cream and VM is recommended for reducing the severity of AVF cannulation pain. *Trial Registration*. Iranian Registry of Clinical Trials, Trial No : IRCT20120109008665N12, registered on 3 June 2020.

## 1. Introduction

Patients with end-stage renal disease (ESRD) need alternative kidney treatments such as hemodialysis (HD), peritoneal dialysis, or kidney transplantation to survive [1].

Among these treatments, HD is the primary choice for patients [2,3]. Successful HD requires vascular access, which can be accomplished through an arteriovenous graft, a central venous catheter, an external arteriovenous shunt, or an arteriovenous fistula (AVF) [4]. Fistulas have a long-term success rate and greater survival among all vascular access options [5]. AVF can be considered the gold standard for vascular access in dialysis patients [6]. Currently, the National Kidney Foundation of the United States has launched a “Fistula First” initiative for vascular access [7] that strongly recommends AVF.

Dialysis requires the use of at least one, and more commonly, two large gauge needles [8]. Since these patients usually experience two cannulations in their fistula three times per week, they experience needle pain at least 300 times a year [9,10].

Pain is an unpleasant feeling and an emotional experience that is associated with potential or actual tissue damage [11]. The pain and stress of AVF puncture with the release of adrenaline can lead to side effects such as fear, irritability, confusion, delayed immune function, and impaired emotional relationships. The short-term side effects of pain also include decreased oxygenation, hemodynamic instability, and increased intracranial pressure [12].

Harris et al. suggested that more than one-fifth of hemodialysis patients are unable to tolerate the pain caused by vascular needles [13] and 47% are afraid of vascular needles [14] and consider vascular needle insertion the most stressful part of the treatment and the biggest concern when undergoing HD [15].

The prevalence of pain due to vascular needle insertion in HD patients has been reported with similar rates in almost all studies. For instance, Alzaatreh et al. in Jordan reported this prevalence as 57%, Kortobi et al. in Morocco as 40–60%, and Da-Silva et al. in Brazil as 58.5% [16–18].

Pain control during vascular needle insertion in HD patients can be important for two reasons: First, pain adversely affects the patients' quality of life [19]; second, it can lead to noncompliance with the treatment regimen (dialysis) [10] and ultimately lead to the patients' death. Therefore, reducing the pain experienced due to vascular needle insertion in HD patients can be very helpful in improving their quality of life and facilitating their treatment acceptance [8].

Among health care personnel, nurses play a key role in reducing patient's pain. Nurses typically use two approaches to reduce the pain of AVF insertion in HD patients: Pharmaceutical and nonpharmaceutical approaches [20]. The effectiveness of some of these methods, however, is still debatable [21].

In Iran, there is usually no single method to relieve AVF cannulation pain in HD patients [22]. Pain relief methods reported in the literature include lavender aromatherapy, massage therapy, rhythmic breathing, relaxation techniques, mental distraction, piroxicam gel and ethyl chloride spray, lidocaine spray, EMLA cream, and using the Valsalva maneuver [23–28].

EMLA cream is a topical anesthetic combination of the two topical anesthetics lidocaine 2.5% and prilocaine 2.5% [29]. By changing the depolarization of cell membranes to sodium ions, this cream blocks the conduction of nerve impulses and can provide analgesia in the superficial layers of the skin to a depth of 5 mm [30,31]. A meta-analysis performed by Fetzer et al. on 20 studies recommended the use of EMLA cream for VP because of its potential to reduce pain [32]. In another study, Mirzaei et al. stated that this cream is effective in reducing pain caused by AVF cannulation in HD patients [33]. Rogers et al. however, reported that EMLA cream might not have any pain relief effect when seeking access to blood vessels in affected children and might require more attention from the nurse [34]. Mohseni et al. investigated the effect of the two topical medications piroxicam and EMLA in comparison with placebo on pain severity caused by fistula cannulation in HD patients and found that EMLA cream significantly reduced needle insertion pain compared to piroxicam gel and placebo (*P* < 0.001) [30]. The use of EMLA cream in HD patients also causes hemodynamic stability at the start of HD by the reduction of pain during AVF cannulation [35].

Kortobi et al. also studied the effect of cryotherapy in comparison with EMLA cream in the management of AVF puncture pain. The results showed that the analgesic effects of both techniques were approved, but cryotherapy provided higher efficiency and fewer restrictions for patients and could be suggested for AVF puncture pain management [17].

The Valsalva Maneuver (VM) is also a noninvasive method for controlling AVF puncture pain [36]. This method was first introduced by an Italian physician named Antonio Maria Valsalva in 1704 as a forced exhalation against a closed airway. This maneuver stimulates pressure receptors in the carotid sinus, aortic arch, and cardiopulmonary baroreceptors by increasing the pressure inside the chest and abdomen; by stimulating the vagus nerve, this maneuver ultimately inhibits the spinal pain receptors and prevents the transmission of pain impulses [37]. VM can also be performed by blowing into a rubber tube connected to a mercury barometer and bringing its pressure to 20 mm Hg [38]. Performing VM does not require any equipment, is easy for the patients to learn, reduces the pain severity caused by peripheral venous cannulation, and also increases the success of venous cannulation [39]. One study has shown that using VM can reduce pain when inserting a needle into the fistula of HD patients [39]. Soltani Mohammadi et al. and Kumar et al. (2016) showed that the pain caused by skin puncture in spinal injections is reduced by performing VM [40,41]. Similarly, Nazemroaya et al. confirmed the effect of this maneuver on reducing pain during general anesthesia [42]. This maneuver causes distraction and is therefore used to relieve the pain caused by venipuncture in children [43]. In another study, Babaei et al. also reported the positive effect of VM in reducing pain and hemodynamic changes during venous cannulation [44]. Another study confirmed the significant reduction in pain severity with the use of VM during AVF insertion compared to controls (*P* < 0.001). PerformingVM for 16–20 seconds during needle insertion into a vein can reduce the incidence and severity of pain experienced by the patient [20].

Although many studies have shown the effectiveness of EMLA cream [30,32,33] and VM [39–44], the two techniques have not been compared in terms of their capacity to reduce AVF cannulation pain in HD patients. Given that the management of symptoms (especially pain) is a top priority for patients with ESRD [45] and due to the contradictory findings on the effectiveness of EMLA cream and VM in some studies, the need to choose the best method to control these patients' pain, and the lack of knowledge about whether EMLA relieves pain better or VM, this study was conducted to compare the effectiveness of EMLA cream and VM on pain severity during vascular needle insertion in HD patients using superiority testing procedures.

## 2. Methods

### 2.1. Study Design and Participants

**Figure 1:**
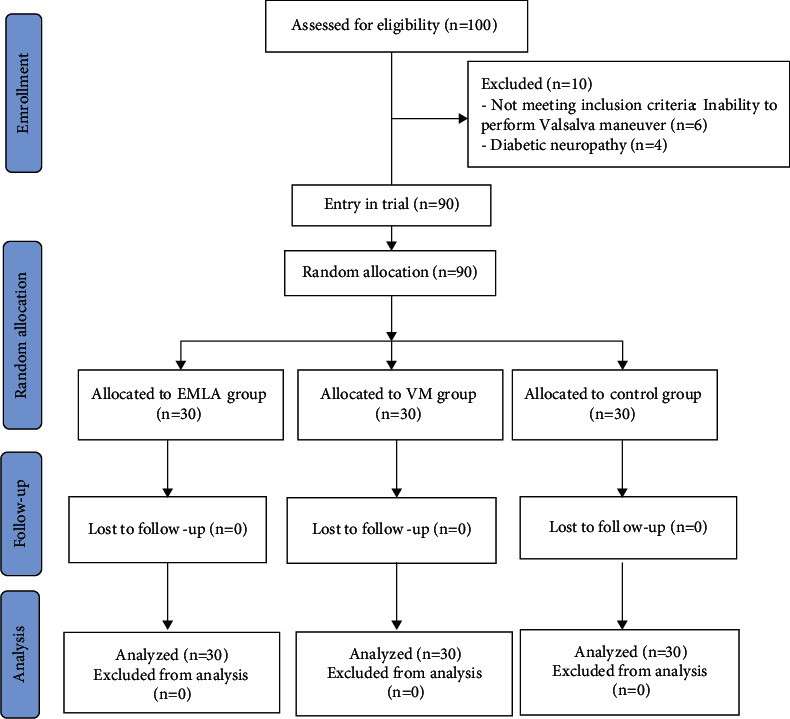
Presents the flow chart of the participants. Figure 1 CONSORT flowchart of the study.

This randomized, parallel, clinical trial without blinding was carried out from July to October 2020 on HD patients admitted to the hemodialysis ward of Kowsar Hospital, affiliated with Semnan University of Medical Sciences, in Semnan, Iran. Similar studies [30,39] were used to determine the sample size. Since the sample size for EMLA cream has been proposed as 25 with mean pain severity and standard deviation of 1.36 ± 1.22 [30], and as 30 with mean pain severity and standard deviation of 2.4 ± 0.7 for VM [39], considering 95% confidence interval and 80% test power and using the following formula (*n*=((*z*_*1-α/2*_* + z*_*1-*_*β*)2×(*δ*12 + *δ*22))/(*μ*1-*μ*2)2), the sample size was taken as 30 per group. The effect size was estimated as1.04 in G^*∗*^power software. A total of 100 people were recruited for the study. Ten of them were excluded for various reasons and the analysis was finally performed on 90 patients (30 subjects per group). The eligible patients were randomly assigned to one of either control, VM, or EMLA cream groups (Figure 1). To assign the patients to the three groups, sealed envelopes containing the letters E (EMLA cream), V (Valsalva maneuver), and C (control) were used and every patient chose their own envelope. Because patients using VM or EMLA cream were easily identifiable and the researchers had to instruct the patients on how to use EMLA cream and perform VM, blinding was not possible. Therefore, the present study was performed with an open-label design.

The inclusion criteria were age over 18 years, at least 3 months experience of HD, ability to read and talk, no addiction or dependence on painkillers, no pain before cannulation, no pain in the last 24 hours, no fistula ulcers, no history of skin allergies or dermatitis, no use of Phenobarbital, sulfonamides, or barbiturates, no issues in accessing the blood vessels, not having a pacemaker, no history of liver disease, diabetic neuropathy, peripheral vascular disease, dementia, or Alzheimer's disease, no respiratory or brain disease, glaucoma, or increased ICP, and no history of recent eye surgery. The exclusion criteria were the patient's inability to perform VM and keep the mercury column above 20 mm Hg for 16–20 seconds, unsuccessful cannulation on the first try, known myocardial infarction or dangerous hemodynamic disorders or arrhythmias, and unwillingness to continue participation in the research for any reason.

### 2.2. Ethical Considerations

Ethical considerations of research were taken into account when conducting this study. The research protocol was approved by the ethics committee of Semnan University of Medical Sciences (IR.SEMUMS.REC.1399.061) and was registered in the Iranian Registry of Clinical Trials (IRCT20120109008665N12). The researchers started collecting the data after obtaining permission from the officials of Kowsar Hospital and the hemodialysis unit. In a meeting before the intervention (introduction session and group allocation of the patients), the researchers visited the hemodialysis unit, introduced themselves to the patients, and gave sufficient explanations about the research objectives and work method, confidentiality of the data, and the voluntary nature of participation in the study. The patients' questions were also answered and written consent was obtained from them to participate in the study.

### 2.3. Interventions

In the introduction session, the patients in the EMLA cream group were given a 5-gram EMLA cream of 5% (AstraZeneca, Eczacibasi Ltd., Lüleburgaz, Turkey) and a transparent dressing and were instructed to apply half of the cream to the area on their skin where the needle would be inserted and cover it with a transparent dressing without rubbing the area, one hour before the start of their next dialysis session, at home. The patients in the VM group were instructed on how to blow into a disposable plastic tube connected to a mercury barometer (Easy Life, TXJ-10B model, China) to the point that the mercury column would raise to 20–25 mmHg for 16–20 seconds. No special intervention was performed on the control group and they received only routine care measures.

In the next dialysis session, in the EMLA group, after removing the transparent dressing and cleaning the skin, the area was disinfected with 70% alcohol-soaked cotton. AVF cannulation was performed and fixed by the patient's dedicated nurse and the patient's pain was measured using the visual analog scale (VAS) two minutes after arterial needle insertion.

In the VM group, the patients were asked to blow in a plastic tube connected to a mercury barometer after taking a deep breath, so that the mercury column would raise to 20–25 mmHg for 16–20 seconds. After performing VM and after disinfection of the area with 70% alcohol-soaked cotton, AVF cannulation was performed and fixed by the patient's dedicated nurse and their pain was measured using VAS two minutes after arterial needle insertion.

In the control group, after disinfecting the area with 70% alcohol-soaked cotton, AVF cannulation was performed and fixed by the patient's dedicated nurse and their pain was measured using VAS two minutes after arterial needle insertion.

To ensure the homogeneity of the procedure in all three groups, a skilled hemodialysis nurse inserted a hemodialysis needle No. 16 (Wing Eater, Singapore) into the veins from a distance of at least 5 cm from the fistula at a 30–45° angle with the bevel of the needle facing up.

It should be noted that since local anesthesia occurs 60 minutes after applying EMLA cream depending on the site, cannulation was performed 60 minutes after using EMLA cream [46].

### 2.4. Data Collection

The data collection tools in this study were a demographic information questionnaire and VAS. The demographic information questionnaire included items such as age, sex, marital status, education, the underlying cause of CRF, time since the start of dialysis, duration of dialysis sessions, and frequency of dialysis per week.

The patients' pain severity was assessed using VAS on a scale of 0 to 10. The patients were asked to choose a number from 0 (no pain) to 10 (the most severe pain imaginable) for the severity of pain experienced after the needle was inserted. On this scale, pain severity is divided into four groups: painless (0), mild pain (1–3), moderate pain (4–7), and severe pain (8–10) [47]. Alghadir et al. approved the reliability of this scale with an intracluster correlation coefficient of 0.97 [48]. In the present study, the reliability of VAS was confirmed with Cronbach's alpha of 0.96 based on a pilot study on ten patients.

### 2.5. Statistical Analysis

Data analysis was performed using the perprotocol approach in SPSS-19 statistical software (SPSS Inc., Chicago, IL, USA) at a significance level of 0.05. By drafting the absolute and relative frequency tables, the research data were described, categorized, and compared. To analyze the data, first, the Kolmogorov‒Smirnov test was used to determine the normal distribution of data. The Chi-square test was then used to compare the absolute and relative frequency of the subjects in terms of gender, the underlying cause of CRF, duration of each dialysis session, and frequency of dialysis per week. The ANOVA was used to compare the absolute and relative frequency of the subjects in terms of age and duration of dialysis. The Kruskal–Wallis test was used to compare the mean pain severity experienced by the subjects during AVF cannulation, and the Mann‒Whitney post-hoc *U* test was administered for the pairwise comparison of the mean pain severity during vascular needle insertion in the three groups.

## 3. Results

### 3.1. Participants' Characteristics

The present study was conducted on 90 patients undergoing hemodialysis. The mean age of the patients was 54.11 ± 13.49 years. Most patients in the EMLA cream and VM groups (76.7%) and 63.3% of the patients in the control group were males.  Table 1 shows the demographic and disease-related data of the subjects. The three groups were not significantly different in terms of demographic and disease-related variables (*P* > 0.05).

### 3.2. Severity of Pain

As for the absolute and relative frequency distribution in terms of pain severity during AVF cannulation, the results showed that the pain severity of the majority of subjects was mild in the EMLA (40%) and VM (46.7%) groups but moderate (60%) and severe (40%) in the control group. In terms of pain severity during AVF cannulation, the lowest mean pain severity pertained to the EMLA cream group (2.06 ± 2.19) and the highest mean pain severity to the control group (6.20 ± 1.49). The mean pain severity in the VM group was 3.2 ± 30.42. The Kruskal‒Wallis test showed a significant difference in pain severity during AVF cannulation between the three groups (*P* < 0.001) (Table 2).

The complementary Kruskal‒Wallis test (Mann‒Whitney *U* test) for the pairwise comparison of the mean pain severity of the groups showed that the pain severity differed significantly between the EMLA vs. control (*P* < 0.001) and VM vs. control group (*P* < 0.001), but no significant difference was found between the EMLA and VM groups themselves (*P*=0.067) (Table 3).

## 4. Discussion

The results of the present study, which was conducted to compare the effect of EMLA cream and VM on pain severity during AVF cannulation in HD patients showed that EMLA cream and VM provide similar pain relief. To the best of the researchers' knowledge, no clinical trials have compared the pain-relieving effects of EMLA cream and VM on AVF cannulation to date.

Compared to the controls, the mean pain severity after cannulation was significantly lower in the EMLA group, followed by the VM group. The results showed a significant difference in pain severity between the three groups. The pairwise comparison of the mean pain severity also showed a significant difference in pain severity between the EMLA cream and control groups and between the VM and control groups. No significant difference was found between the EMLA and VM groups.

In line with this finding, several other studies have been conducted to investigate the effect of EMLA cream or VM in reducing pain caused by venipuncture and AVF cannulation, confirming the effectiveness of these methods [20,33,49–51]. Regarding EMLA cream, the results of a study by Suren et al. to evaluate and compare the effect of VM and EMLA on the severity of venipuncture pain in three groups of VM, EMLA, and control showed that VM had similar results to EMLA in terms of venipuncture pain relief, which is consistent with the present findings. Therefore, both VM and EMLA can be used to reduce the objective and subjective pain caused by venipuncture [20]. The results of a double-blind randomized study conducted by Fujimoto et al. on 66 patients undergoing chronic hemodialysis to compare the effect of EMLA cream with lidocaine tape for AVF cannulation showed EMLA cream to be much more effective on VAS scale (VAS) (*P*=0.00001) compared to the lidocaine tape [49]. The results of a study by Benini et al. examining the effect of EMLA cream on AVF cannulation pain control in children during hemodialysis showed that EMLA is effective in reducing pain in these children [50]. The results of a study by Mirzaei et al. comparing the effectiveness of EMLA cream with lidocaine spray and ice pack in reducing the severity of AVF cannulation pain also showed that the mean pain reduction was significantly higher in the EMLA group compared to the lidocaine spray and ice pack groups [33]. A study on the use of EMLA cream to reduce phlebotomy pain in adults showed that phlebotomy pain is significantly reduced even just 5 minutes after applying EMLA cream [51]. Contrary to this finding, the results of a study by Kortobi et al. on the effect of cryotherapy compared to EMLA cream in the management of AVF cannulation pain showed that cryotherapy offers greater efficiency and fewer restrictions for patients [17]. The results of a study by Qane et al. on the effect of EMLA cream on the pain caused by lumbar puncture showed that the use of this cream is not effective in reducing this type of pain [52]. The reason for this discrepancy can be attributed to the nonuse of dressings to cover EMLA cream in the study by Kortobi et al., the deeper penetration of a lumbar puncture needle, and the superficial effect of the medication in the study by Qane et al.

Several studies on VM have also confirmed the effectiveness of this nonpharmacological method in reducing pain severity. The results of a study by Vijay et al. to investigate the effect of VM on the severity of peripheral venous cannulation pain showed that the mean score of subjective pain was significantly lower in the VM group compared to the control group [53]. In some studies, VM has been offered as a simple, inexpensive, and effective method to reduce venipuncture pain without any side effects [38,54]. Mohammadi et al. used VM in their study to reduce the pain caused by lumbar puncture [55]. Sundaran et al. and Suren et al. showed that VM can be used to reduce patients' pain during intravenous puncture [20,36]. The findings reported by Saputra showed a significant difference in pain severity during AVF cannulation before and after performing VM in HD patients [56].

The relief of the pain due to AVF cannulation in hemodialysis patients can be caused by the activation of the cardiopulmonary baroreceptor reflex arc or the Sino aortic baroreceptor arc following VM. Activation of the baroreceptor can stimulate the vagus nerve. The activated vagus nerve then sends impulses to the nucleus of the solitarius tract. The solitarius tract is the point of intersection between the afferent nerves of the nervous system and the nociceptive pain pathways in the spinal lamina [57]. Pain relief during AVF cannulation occurs when the solitarius tract first receives a stimulus impulse from vagus nerve activation due to VM. At the same time, the stimulation of the pain transmitted by the nociceptive nerve as a result of AVF cannulation, which also passes through the solitary tract, is inhibited by the impulses sent by the vagus nerve. Therefore, the pain felt by the patient is reduced or disappears. This process is consistent with the gate control theory of pain proposed by Melzack and Wall [58].

Regarding the mean pain severity, the results did not show a statistically significant difference between the EMLA (2.06) and VM (3.30) groups. This difference was, however, clinically significant. Only one study compared the severity of pain after venipuncture following the use of EMLA cream and VM, which yielded consistent results with this study [20].

In the present study, side effects such as localized pallor were observed in ten patients following the use of EMLA cream, while side effects such as bradycardia, hypotension, or fainting were not observed during or after VM.

According to the researchers, before proposing EMLA or VM as a preferred method to reduce the severity of pain in patients during AVF cannulation, the following points should be considered. The use of EMLA cream is not only limited by specific considerations, mainly related to its duration of action (30–60 minutes) and contraindications (congenital methemoglobinemia, porphyria, and hypersensitivity to one of its components), but will likely cause some side effects as well (erythema, itching, burning sensation, purpura, edema at the site of application, or even contact dermatitis). EMLA cream is also not readily available in many countries, including Iran, and is not economically viable for most patients (€35 per month, to be applied three times a week) [20]. In contrast, the advantages of VM include ease of implementation, patient operability, low cost, rare side effects, and, most importantly, being nonpharmacological [39].

This study confirmed the hypothesis that EMLA cream is clinically superior to VM, although no statistically significant difference was observed between the two approaches.

### 4.1. Study Limitations

One of the most important limitations of the present study was the decrease in the internal validity of the study due to its lack of blinding. Pain is a subjective phenomenon that is reported by the individual and every person has a different opinion about the severity of pain they experience, which can lead to the over or underestimation of pain. Further studies with larger sample sizes and the use of objective criteria for assessing pain severity, blood pressure, and heart rate monitoring before and after cannulation, and measuring the serum levels of lidocaine and prilocaine to determine the safety of EMLA cream up to 90 minutes after use are some of the measures recommended obtaining more precise evidence on the effectiveness of VM and EMLA cream in reducing the severity of AVF cannulation pain.

## 5. Conclusion

Based on the results, VM is as effective as EMLA in reducing the pain caused by AVF cannulation. Due to the importance of the management of pain as the fifth vital sign, and the side effects of its poor control, the use of VM and EMLA cream is recommended for reducing the severity of pain caused by AVF cannulation in HD patients. Due to the lack of easy access to EMLA cream and the problems with pharmaceutical methods, using VM as an independent complementary/alternative nursing measure is recommended.

## Figures and Tables

**Table 1:** The demographic and disease-related characteristics of the participants in the EMLA, VM, and control groups.

Groups Characteristics	EMLA (*n* = 30) N (%)	VM (*n* = 30) N (%)	Control (*n* = 30) N (%)	*P* value^*∗*^
Gender Male Female	23 (76.7) 7 (23.3)	23 (76.7) 7 (23.3)	19 (63.3) 11 (36.7)	*X* ^2^ = 1.77, 2 *p*=0.412

Age (years) <40 40–60 >60	7 (23.3) 13 (43.3) 10 (33.3)	5 (16.7) 19 (63.3) 6 (20)	2 (6.7) 19 (63.3) 9 (30)	*X* ^2^ = 5.16, 4 *p*=0.271

Underlying disease Diabetes HTN Diabetes + HTN etc.	6 (20) 4 (13.3) 7 (23.3) 13 (43.3)	9 (30) 6 (20) 7 (23.3) 8 (26.7)	10 (33.3) 4 (13.3) 7 (23.3) 9 (30)	*X* ^*2*^ *=* *3.01, 6* *p*=0.807

Duration of HD (month) <12 12–24 25–36 37–48 >48	3 (10) 7 (23.3) 4 (13.3) 3 (10) 13 (43.3)	4 (13.3) 3 (10) 3 (10) 5 (16.7) 15 (50)	3 (10) 8 (26.7) 5 (16.7) 2 (6.7) 12 (40)	*X* ^2^ = 4.78, 8 *p*=0.780

Duration of HD sessions (hour) 2 3 4	2 (6.7) 4 (13.3) 24 (80)	0 (0) 3 (10) 27 (90)	1 (3.3) 10 (33.3) 19 (63.3)	*X* ^2^ = 8.45, 4 *p*=0.076

HD sessions per week (times) 2 3 4	5 (16.7) 22 (73.3) 3 (10)	5 (16.7) 25 (83.3) 0 (0)	8 (26.7) 21 (70) 1 (3.3)	*X* ^2^ = 4.88, 4 *p*=0.30
	(Mean ± SD)	(Mean ± SD)	(Mean ± SD)	*P* value^*∗∗*^

Age (years)	53.10 ± 15.92	52.30 ± 11.79	56.93 ± 12.40	*F*(2,87) = 1.01, *p*=0.368

Duration of HD (month)	50.90 ± 34.48	67.43 ± 51.92	55.76 ± 49.02	*F*(2,87) = 1.03, *p*=0.360

EMLA : Eutectic Mixture of Local Anesthetics; VM : Valsalva maneuver; HD : Hemodialysis; HTN : Hypertension ^*∗*^Chi-square test; and ^*∗∗*^One-way ANOVA test.

**Table 2:** Distribution of absolute, relative frequencies and mean scores of the research units according to the severity of pain during AVF cannulation in the EMLA, VM, and control groups.

Groups Severity of pain	EMLA (*n* = 30 N (%)	VM (*n* = 30) N (%)	Control (*n* = 30) N (%)	*P* value
No pain (0)	11 (36.7)	3 (10)	0 (0)	^*∗*^ *X* ^2^ = 45.17, 6 *P* < 0.001
Mild (1–3)	12 (40)	14 (46.7)	0 (0)	
Moderate (4–7)	6 (20)	8 (26.7)	18 (60)	
Severe (8–10)	1 (3.3)	5 (16.7)	12 (40)	
Mean pain score (Mean ± SD) 95% CI^*∗∗∗*^	2.06 ± 2.19 1.24–2.88	3.30 ± 2.42 2.39–4.20	6.20 ± 1.49 5.64–6.75	^*∗∗*^ *X* ^2^ = 36.02, 2, *P* < 0.001

^*∗*^Chi-square test; ^*∗∗*^ Kruskal‒Wallis test; ^*∗∗∗*^Confidence interval.

**Table 3:** Pairwise comparison of mean scores of the pain severity in the EMLA, VM, and control groups.

Groups	Value	*p* value^*∗*^	95% CI^*∗∗*^
EMLA-VM	−1.23	0.067	(−2.54)−(0.75)
EMLA-control	−4.13	<0.001	(−5.44)−(−2.82)
VM-control	−2.90	<0.001	(−4.20)−(−1.59)

^*∗*^Mann‒Whitney *U* test; ^*∗∗*^Confidence interval.

## Data Availability

All data generated or analyzed during this study are included within the article.
